# Static Mixers Producible by Additive Manufacturing: Novel Rapid Automatic Optimisation and Practical Evaluation

**DOI:** 10.3390/polym14214646

**Published:** 2022-11-01

**Authors:** Jana Sasse, Malte Schön, Christian Hopmann

**Affiliations:** Institute for Plastics Processing (IKV) in Industry and Craft, RWTH Aachen University, Seffenter Weg 201, 52074 Aachen, Germany

**Keywords:** static mixer, extrusion, OpenFOAM, optimisation, additive manufacturing

## Abstract

In the extrusion of plastics, the thermal and material homogeneity of the plastic melt at the die entry are of high importance for the extrudate quality. While static mixers are widely used to improve the melt homogeneity, previous attempts at optimisation for reduced pressure loss and improved mixing had to be performed by hand and human experience, limiting the degrees of freedom and efficiency. A new automatic optimisation method based on the open source software OpenFOAM was developed. Using immersed boundary methods, new target functions in the pre-existing routine *adjointShapeOptimizationFoam* and an additional algorithm checking the suitability for additive manufacturing and fixing the geometry during run-time is presented. The new algorithm is used to optimise an existing static mixer based on an X-type geometry with integrated oil channels, maximising the heat exchange between oil and melt. Based on the results of these simulative optimisations, the best candidates were manufactured using selective laser melting and experimental trials were run. Experimental validation shows that with our optimisation algorithm, a pressure loss reduction of 10% could be achieved. The core melt temperature was reduced by 6 ${}^{\circ }$C, improving the thermal homogenisation as well. While the main advantage of this method is the rapid optimisation taking the operating point into account, the trials also showed positive results in off-design operating points. This allows the low-cost design and manufacture of individualised static mixers.

## 1. Introduction

In the extrusion of plastics, the thermal and material homogeneity of the plastic melt at the die entry are of high importance. Thermal inhomogeneities can be introduced, e.g., by high dissipation and lead to thickness variations over the outlet in the extrudate [1]. Material homogeneity on the other hand is vital in foam extrusion and other applications, where a uniform distribution of blowing agents, colourants, additives and processing agents is required. In order to avoid production scrap, static mixers are used to improve homogeneity by splitting and redistributing the melt flow. Static mixers are also used in other applications, such as the synthesis of polymers [2]. There are different types of static mixers available, and mixers of the X-type are widely used as their geometry provides numerous flow divisions and recombinations on a short length.

Most static mixers on the market are manufactured using conventional methods. On the other hand, additive manufacturing (AM), in particular selective laser melting (SLM), allows for new types of geometries for components of extrusion lines due to the added degrees of freedom [3]. In SLM, the geometry is printed in slices. First, a thin layer of metal powder is added on the substrate platform. Using a laser, the powder is melted in places and thus fused with the layers below. After cooling, the substrate platform is lowered, a new layer of metal powder is added and the next layer can be fused to the additively manufactured part. As the metal powder provides only limited support and heat extraction for upper layers, support structures must be added to the print in locations where critical overhangs are present [4]. When the print is finished, the support structures are removed and the print is post-processed (usually sandblasted), removing the remaining powder and improving the print’s surface quality. Typical minimal layer thicknesses range from 20–60 μm, depending on the surface quality requirements and material [5]. The unfused metal powder can be retrieved and reused for future prints, making SLM a method of manufacture with little material waste. It is relatively affordable, fast and cost-efficient in small batch sizes, making it ideal for individualised applications [6].

Because SLM is fast and cheap, we can for the first time consider making “bespoke” individualised static mixers instead of a “one size fits all” solution. However, determining a suitable mixer geometry for each application in lab trials is prohibitively expensive. Simulations using Computational Fluid Dynamics (CFD) can be used to evaluate the mixing performance at far lower cost [7]. Different types of parameterised optimisation methods have been utilised for the optimisation of (dynamic) mixing elements. An investigation by Janßen et al. used an automated process to find the local optimum within a parameterised version of a dispersive mixer [8], while Hube et al. used a combination of free-form deformation and surface splines to parameterise and optimise rhomboid mixing elements, using a linear-elasticity-based mesh update method to deform the mixing element without remeshing [9]. Within the category of X-type static mixers, different configurations and designs are possible and subject to optimisation. Among the degrees of freedom of a static mixer are not only the number of mixing elements, but also the number of cross-bars over the width of the channel $N_{x}$, the number of parallel cross-bars per mixing element $N_{p}$ and the angle between the bars θ. Previous research has shown that an optimal configuration for any X-type static mixer can be established via the design rule $N_{p}=\left(2/3\right)N_{x}-1$ for $N_{x}=3,6,9,\cdots$ [10,11]. It has also been shown that the X-type static mixer design is superior to most other static mixer designs, as it maximises mixing performance per pressure loss [12]. However, the design of static mixers is marked by the high amount of degrees of geometric freedom, as the parameterisation method described above only applies to symmetrical mixers with rectangular bars.

Apart from mixing, another goal can be the cooling of the melt. Conventional static mixers introduce considerable viscous dissipation as the melt is subjected to significant shear and elongation by the mixing bars. To counter-balance that, integrated oil channels enable simultaneous mixing and cooling of the melt, like in the P1 cooling mixer (Promix Solutions AG, Winterthur, Switzerland) [13].

This cooling transforms the mixing task from a somewhat isothermal flow problem to a heat transfer problem, often necessitating the use of simulation software. Past simulations of other types of static and dynamic mixers have been performed in the open source software OpenFOAM (OpenFOAM Foundation Ltd., London, UK) by Erb et al. [14], Alexias et al. [7,15] and Kettemann et al. [16]. In order to model material mixing, i.e., the transport of matter, 3D advection-diffusion equations can be used to model the transport of a passive scalar. While complex geometries such as the X-type static mixer are challenging even for state-of-the-art meshing softwares, the use of Immersed Boundary (IB) methods allows us to circumvent this problem. In IB methods, the whole computational domain is meshed as one domain, and subsequently, parts of the mesh are defined as belonging to the solid domain and made impassable to flow. This allows not only for a computationally cheap way of meshing the geometry, but also avoids re-meshing when the geometry changes. The OpenFOAM module *adjointShapeOptimizationFoam*, which was first introduced by Othmer et al. [17,18], takes advantage of this implementation. In this module, an adjoint optimisation algorithm using a gradient-based approach adjusts the boundaries between the fluid and solid domain by changing the extent of the solid domain according to local information on which parts of the domain are favourable or counterproductive for the overall target. While the original implementation was targeted towards minimal pressure loss of the fluid, other target functions are also possible. Previous research not published within the widely-shared OpenFOAM repositories includes the homogeneity of a passive scalar at the outlet [19,20] and also thermal optimisation models, e.g., with the goal of reaching a homogeneous temperature distribution or a specified target temperature [21,22,23,24]. While these optimisation algorithms yield good results, geometries that are compatible with additive manufacturing require additional checks and modifications [25].

**Figure 1:**
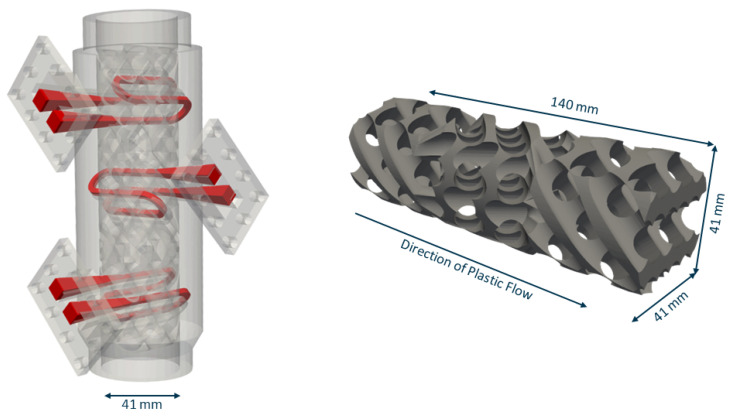
(**Left**) Static mixer with integrated temperature control channels. Adapted with permission from Ref. [28]. 2020, Institute for Plastics Processing (IKV) at RWTH Aachen. (**Right**) Static mixer suitable for SLM based on an X-type mixer geometry.

In previous work at the Institute for Plastics Processing (IKV) at RWTH Aachen, an OpenFOAM simulation environment has been developed specifically for the simulation of plastic melt in extrusion processes. This includes the implementation of shear rate- and temperature-dependent material models, the integration of a 3D energy equation and a 3D advection-diffusion equation, as well as an immersed boundary method with material dependent specific heat capacities and diffusion coefficients [26,27,28,29,30]. This simulation environment has already been applied and validated in lab trials for the application and manual optimisation of static mixers [26,27,28,29]. In some of these applications, additive manufacturing has been successfully applied to introduce a higher degree of freedom [9,26,28,29]. In Figure 1 the resulting static mixer geometry of this research [26,27,28] is shown, where an X-type mixer has been modified to satisfy SLM manufacture requirements. In addition, three temperature control channels have been integrated. It has been shown that this static mixer geometry improves the ratio of thermal mixing and pressure loss by 50% and enables cooling of the melt by up to 10 ${}^{\circ }$C, which is why we use it as the basis of the optimisation in this paper.

While the results shown above have been promising, the design and optimisation of this static mixer was largely crafted by hand and is therefore very inefficient, as it requires a large amount of skilled labour. The objective, then, is not only the automatic design of improved static mixers for plastics extrusion, but also the reduction of development time for new geometries. The goal of the research presented is the automatic optimisation of static mixers in OpenFOAM. Based on the module *adjointShapeOptimizationFoam*, new target functions are introduced specific to the optimisation of static mixers. In an additional step, the algorithm checks with each update of the mixer geometry whether the new geometry is additively manufacturable and automatically adjusts the geometry if necessary. This new algorithm is used to optimise a version of a static mixer with integrated oil channels for additional cooling using different target functions and oil temperatures, and the most promising results are manufactured using SLM and practically evaluated in lab trials.

## 2. Materials and Methods

First, a numerical model for the simulation and optimisation of the static mixers was developed. The presented model is specific to the needs of polymers, such as their high viscosity and strictly laminar flow regimes with according shear heating and simultaneous low heat capacity. Subsequently, optimisations were performed on pre-existing static mixer geometries which include integrated oil channels and influencing the optimisation routine. The best candidates were then additively manufactured using SLM and tested under lab conditions.

### 2.1. Numerical Model

While there are different types of IB methods available, one common method is to make the solid domain impassable to flow by introducing an additional term to the momentum equation, which includes an individual porosity α for each cell, which is set to zero for fluid domain cells and to a non-zero value for solid domain cells [17,31].

*adjointShapeOptimizationFoam* is based on a gradient-based optimisation algorithm using Lagrange multipliers [17]. In this algorithm, the state variables are solved in both a primal and an adjoint set of partial differential equations, the combination of which can subsequently be used to compute the sensitivities of the state variables u, *p* and *T*. The topology of the static mixer modelled by the IB method is then optimised using the local sensitivities. As this method allows to compute the complete gradient using only two solver calls (primal and adjoint) and is independent of the search space dimension (i.e., the space spanned by the degrees of freedom), this algorithm is a computationally cheap way to obtain optimised static mixer topologies.

The flow of plastic melt in the static mixer is governed by the Navier-Stokes equations, which for this purpose have been extended by a 3D energy equation and a 3D advection-diffusion equation [30]. The set of primal equations of mass, momentum, energy and diffusion are therefore

(1) ∇·u=0,

(2) $$\nabla p_{norm}=\nabla \cdot \left(u\times u\right)-\nabla \cdot \tau_{norm}-\alpha u,$$

(3) $$0=\nabla \cdot \left(uT\right)+\nabla \cdot \left(D_{T}\nabla T\right)+\frac{\tau_{norm}}{c_{p}}:\left(\nabla \times u\right),$$

(4) $$0=\nabla \cdot \left(uc\right)+\nabla \cdot \left(D_{c}\nabla c\right),$$

where u, $p_{norm}$, *T* and *c* denote the velocity, the pressure normalized to the density ρ, the temperature and the concentration, respectively. In addition, α is the individual porosity introduced by the immersed boundary method. $D_{T}$, $D_{c}$ and $c_{p}$ denote the local thermal diffusivity, the local diffusion coefficient and the local specific heat capacity, while $\tau_{norm}$ is the tensor of the shear stress normalized to the density ρ with ν denoting the kinematic viscosity:

(5) $$\tau_{norm}=\nu \left(\left(\nabla \times u\right)+{\left(\nabla \times u\right)}^{T}\right).$$

The application at hand requires the distinction between three domains: plastic melt, solid steel and oil. Therefore, the material model was extended. Using the parameter $\alpha_{oil}$, the viscosity was set individually for each cell. If a cell was flagged to contain plastic melt, the viscosity was modelled as a shear-thinning non-isothermal fluid using a Carreau-WLF model (see Equations (6) and (7)), while the viscosity for cells containing oil was set using a Newtonian model with a constant viscosity.

(6) $$\nu =\frac{a_{T}\cdot A}{{\left(1+a_{T}\cdot B\cdot \dot{\gamma }\right)}^{C}}$$

(7) $$\mathrm{lg}\left(a_{T}\right)=-\frac{8.86(T-T_{s})}{101.6K+T-T_{s}}$$

The module *adjointShapeOptimizationFoam* was first described in [18]. It uses an immersed boundary method to optimise the shape of a flow canal for minimal total loss of pressure (PtLoss). This code was extended to also include an adjoint 3D energy equation. The resulting set of adjoint equations of mass, momentum and energy are as follows:

(8) $$\nabla \cdot u_{a}=0,$$

(9) $$\nabla p_{a,norm}=\nabla \cdot \left(u_{a}\times u_{a}\right)-\nabla \cdot \tau_{a,norm}-\alpha u_{a},$$

(10) $$0=\nabla \cdot \left(uT_{a}\right)+\nabla \cdot \left(D_{T}\nabla T_{a}\right)+\frac{\tau_{a,norm}}{c_{p}}:\left(\nabla \times u_{a}\right),$$

where the index a denotes the adjoint quantity and the adjoint shear stress tensor $\tau_{a,norm}$ is computed using the adjoint velocity and a viscosity calculated from an adjoint shear rate.

For the optimisation, the target functions for pressure loss and thermal homogenisation are incorporated in the outlet boundary conditions for pressure (Equation (11)), velocity (Equation (12)) and temperature (Equation (13)), where $u_{n}$ and $u_{t}$ refer to the normal and tangential velocity, respectively and $T_{target}$ denotes the target temperature for the homogenisation. The weights $w_{p}$ and $w_{T}$ are used here to turn the cost functions on or off. In order to improve the numerical stability, the second order terms were neglected. For a full derivation of the equations we refer to [20,32].

(11) $$p_{a,norm}{|}_{Outlet}=uu_{a}+u_{n}u_{a,n}+TT_{a}+w_{p}\cdot \left(\left(-0.5u^{2}\right)-u_{n}^{2}\right)$$

(12) $$0=u_{a,n}u_{t}-u_{a,n}u_{a,t}$$

(13) $$0=w_{T}\cdot \left|T-T_{target}\right|$$

After each iteration, the immersed boundary field is updated (Equation (14)) using a gradient-based approach, which uses the local sensitivities, where it is decided for each cell whether a fluid cell is favourable or counterproductive for the overall cost function. In this equation, $\alpha_{0}$ and $\alpha_{max}$ are the individual porosities for fluid and solid, respectively, γ and λ are relaxation parameters, $w_{T}$ is a weight turning thermal optimisation in the field update on or off and $\sigma_{u}=u\cdot u_{a}$ and $\sigma_{T}=T\cdot T_{a}$ denotes the local sensitivities with respect to velocity and temperature, respectively.

(14) $$\alpha_{n+1}=\alpha_{n}\left(1-\gamma \right)+\gamma \cdot \min\left(\max\left(\alpha_{n}+\lambda_{p}\sigma_{u}+w_{T}\lambda_{T}\sigma_{T},\alpha_{0}\right),\alpha_{max}\right)$$

**Figure 2:**
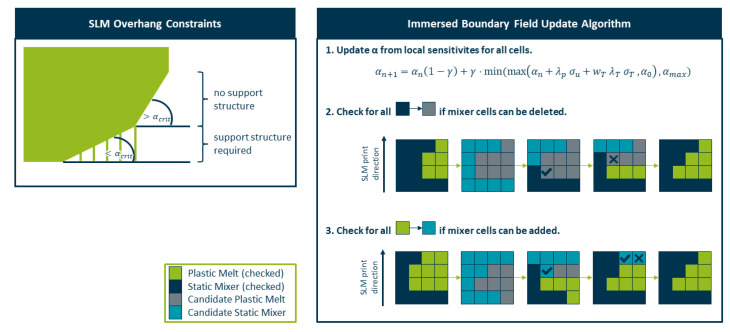
(**Left**) SLM overhang constraints regarding support structures. (**Right**) Schematic of the immersed boundary field update algorithm.

After the immersed boundary field update an additional check is performed to ensure the geometry is still manufacturable using SLM. SLM has two main constraints [4,25,26]:

1. A cross section area of 1 mm${}^{2}$ should be exceeded to prevent leakage and ensure mechanical stability.
2. A critical angle of 45° must be exceeded in every cell (see Figure 2 left). Otherwise support structures need to be added.

**Figure 3:**
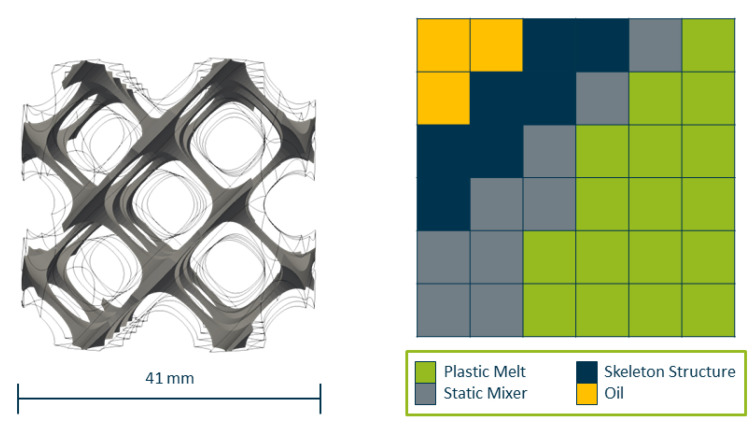
Immersed Boundary field with cells fixed as solid cells belonging to the static mixer or as fluid cells belonging to the oil domain.

Constraint 1 is tackled with the introduction of an additional field $\alpha_{skeleton}$ which provides a minimal mixer structure that cannot be removed by the algorithm (see Figure 3). Cells that are flagged by this variable are automatically set to $\alpha_{max}$ and are exempt from all immersed boundary field updates. Similarly, all cells flagged as belonging to the oil channels by $\alpha_{oil}$ are exempt from the immersed boundary field update, ensuring that they are not removed in the process.

For constraint 2, an additional algorithm is performed after each update (see Figure 2 right). All cells are checked individually in order of their print direction. If a cell changes from solid to fluid, it is checked whether there is a cell in the layer above that requires this cell for support, and if found, the cell has to stay solid. Similarly, if a cell changes its state from fluid to solid, it is checked to determine whether there is a sufficient support structure in the layer beneath, otherwise the change is rejected.

### 2.2. Simulative Optimisation

**Figure 4:**
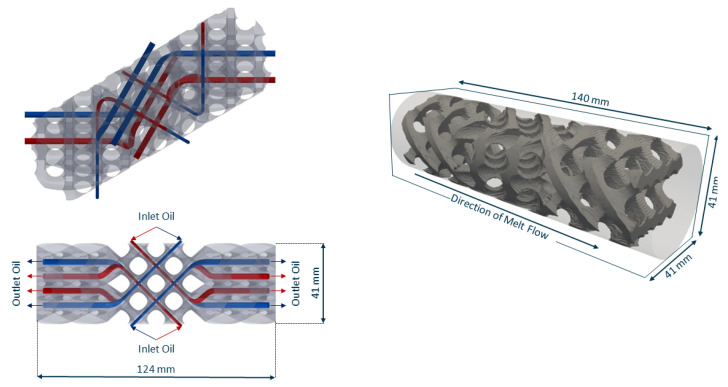
(**Left**) position and direction of flow of oil channels. (**Right**) Computational domain and reference geometry as immersed boundary field.

The reference geometry before optimisation is based on a SLM compatible version of an X-type mixer [26,29]. To maximize the contact area between the oil channels and the melt, a total of 8 oil channels were integrated, allowing for both longitudinal and radial heat exchange (see Figure 4 left).

The whole computational domain (see Figure 4 right) consists of 3.26 million cells. The reference static mixer is comprised of 946,236 cells with 92,773 cells being flagged as $\alpha_{skeleton}$. The oil channels are modelled by 52,451 cells.

**Figure 5:**
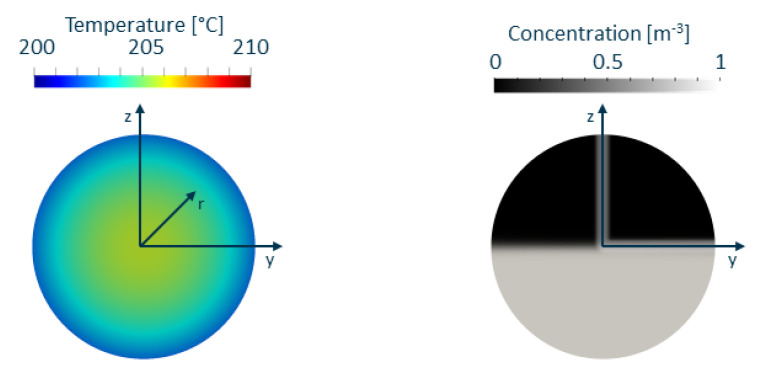
Temperature (**left**) and concentration (**right**) inlet profiles for simulations.

There are different types of thermal inhomogeneities that can typically occur in the extrusion process. One common temperature profile is caused by high dissipation in the extruder [1]; it is visualised in Figure 5 left and mathematically described in Equation (15), where *r* refers to the radius of the profile.

(15) $$T\left(r\right)=\left(-1\times 10^{8}\times r^{4}-44430\times r^{2}+31.615\right)\times 0.17990753+473.15$$

As we intend to implement the experimental method described in [33], a non-uniform inlet concentration profile is used for validation puposes only. The concentration *c* in the advection-diffusion Equation (4) is used to model the transport of a passive scalar by setting the diffusion coefficient $D_{c}$ to a very low value and prescribing a logistic function as a function of a coordinate perpendicular to the profile at the inlet (see Equation (16) and Figure 5 right):

(16) $$c\left(z\right)=\frac{1}{1+\exp(-2000\cdot z)}.$$

The remaining boundary conditions are displayed in Table 1. The $u_{melt}$ was set to a value corresponding to a throughput of 100 kg/h and $T_{melt}$ was set to 200 ${}^{\circ }$C, while $u_{oil}$ was set to a value corresponding to a throughput of 250 L/h for the whole oil circulation system. Previous research has shown that for a throughput of 100 kg/h the pressure loss is about 100 bar. As 20 bar pressure loss corresponds to approximately 1 ${}^{\circ }$C increase in melt temperature and the best homogeneity is achieved if steel temperatures downstream of the screw tip follow this rule [34], the simulations were performed for two oil temperatures, one equal to and one 5 ${}^{\circ }$C higher than the melt temperature.

The three material domains are modelled as follows: The plastic melt is modelled as a non-isothermal shear-thinning fluid with a density ρ = 736 kg/m${}^{3}$, a specific heat capacity $c_{p}$ = 2900 $m^{2}/s^{2}K$, and a thermal diffusivity $D_{T}$ = $1.1997\times 10^{-7}$ $m^{2}/s$, with the Carreau-WLF parameters being A = 12.87 $m^{2}/s$, B = 0.1871 s, C = 0.655 and $T_{s}$ = 237 K. The steel of the static mixer is modelled with a specific heat capacity of $c_{p}$ = 2900 $m^{2}/s^{2}K$ and a thermal diffusivity of $1\times 10^{-5}$ $m^{2}/s$. The oil is modelled with a specific heat capacity $c_{p}$ = 2220 $m^{2}/s^{2}K$, a thermal diffusivity $D_{T}$ = $5.415\times 10^{-8}$ $m^{2}/s$ and a constant viscosity ν = 0.0001 $m^{2}/s$.

First, a full simulation without optimisation was conducted as an initialisation. Subsequently, the optimisation was performed either for minimal pressure loss or for maximal thermal homogenisation. The resulting geometry was then simulated again using the original initial and boundary conditions to ensure that no artificial heating effects occurred in the optimisation process.

### 2.3. Validation Trials

**Figure 6:**
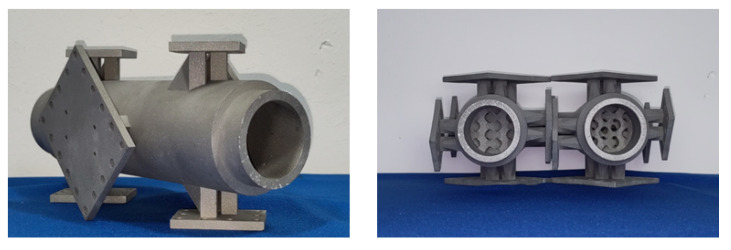
(**Left**) Manufactured static mixer with connectors for oil channels. (**Right**) Reference (**left**) and optimised (**right**) mixer geometries for validation trials.

The resulting geometries were post-processed in Meshmixer (Autodesk Inc., San Rafael, CA, USA), where minor mistakes in the geometry were fixed and the mixer geometry was merged with the outer mixer construction. Subsequently, the static mixers were manufactured using SLM. The resulting static mixers are displayed in Figure 6. The optimised static mixer on the right side differs from the reference static mixer on the left hand side by the decreased thickness of its ‘bars’, suggesting a reduction in pressure loss.

To test the thermal as well as material mixing, a setup with two extruders was used. The validation trials were performed using a 60 mm main single-screw extruder and a 19 mm secondary single-screw extruder for the side feed. For an overall throughput of 20 kg/h, the rotational speed of the main extruder was set to 25 rpm, while the rotational speed of the side extruder was set to 20 rpm. Similarly, a throughput of 80 kg/h was achieved with rotational speeds of 99 rpm in the main extruder and 80 rpm in the side extruder. The material used was a blown film grade of HDPE (Hostalen GD 9550F, LyondellBasell GmbH, Wesseling, Germany), and the side extruder was fed with the same material together with 5% carbon black master batch material.

Both materials were processed at a nominal melt temperature of 220 ${}^{\circ }$C, and the temperatures in the barrel zones were set up as a rising temperature profile (180 ${}^{\circ }$C, 200 ${}^{\circ }$C, 220 ${}^{\circ }$C). All other melt-carrying parts of the setup had a nominal temperature of 220 ${}^{\circ }$C controlled by heating bands. First, trials were run without additional cooling from the oil channels to test the performance of the static mixer alone. For the trials including the oil for additional cooling, the oil temperatures were varied between the nominal melt temperature (220 ${}^{\circ }$C) and a temperature 5 ${}^{\circ }$C higher than the nominal melt temperature (225 ${}^{\circ }$C). Both upstream and downstream of the static mixer, a pressure sensor and an immersed temperature sensor were positioned to measure both the pressure loss of the static mixer and the radial temperature profiles before and after the mixer. The immersed temperature sensor measured the radial melt temperature at r = 0 mm, 7 mm and 14 mm, with r = 20 mm being the temperature at the barrel wall. For more details on the experimental setup, we refer the reader to [33].

For each operating point, 6 samples were taken with a 5 min difference between them to eliminate spontaneous fluctuations in the extrusion process.

### 2.4. Evaluation Criteria

**Figure 7:**
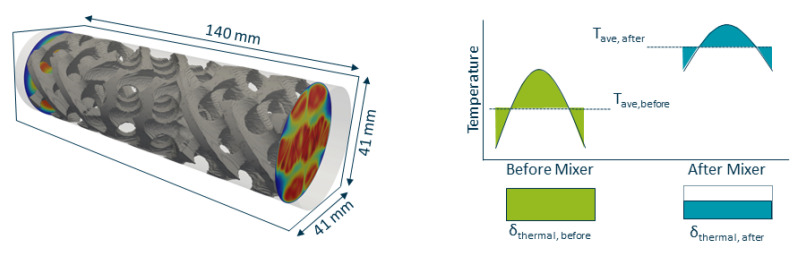
(**Left**) Positions of the cut planes relative to the static mixer for the evaluation of pressure loss. (**Right**) Graphical representation of $e_{thermal}$ and $e_{conc}$ in simulations.

In the simulations, the pressure loss (see Equation (17)) is evaluated using the average value at cut planes before and after the mixer as depicted in Figure 7 left.

(17) $$\Delta p=p_{ave,before}-p_{ave,after}$$

The mixing characteristic numbers $e_{thermal}$ and $e_{conc}$ are defined as in Equations (18) and (19), evaluating the relative change of the cumulative local inhomogeneities (see Figure 7 right). A value equal to zero means no change in the homogeneity and a value equal to one means an ideal maximal homogenisation.

(18) $$e_{thermal}=\frac{\delta_{thermal,before}-\delta_{thermal,after}}{\delta_{thermal,before}}\;\mathrm{with}\;\delta_{thermal}=\sum_{i}\left|T_{i}-T_{ave}\right|,$$

(19) $$e_{conc}=\frac{\delta_{conc,before}-\delta_{conc,after}}{\delta_{conc,before}}\;\mathrm{with}\;\delta_{conc}=\sum_{i}\left|c_{i}-c_{ave}\right|.$$

In the validation trials, the pressure loss is measured using a pressure sensor both before and after the static mixer. In addition, radial temperature profiles were measured both before and after the static mixer and the distribution of black colourant was evaluated as mean grey values $\mu_{grey}$, where a high number is indicative of improved material mixing as a theoretical value of $\mu_{grey}=255$ corresponds to a perfectly black extrudate.

## 3. Results

First, the results of the numerical optimisation are presented and discussed. Subsequently, the results of the validation trials are shown.

### 3.1. Numerical Optimisation

**Figure 8:**
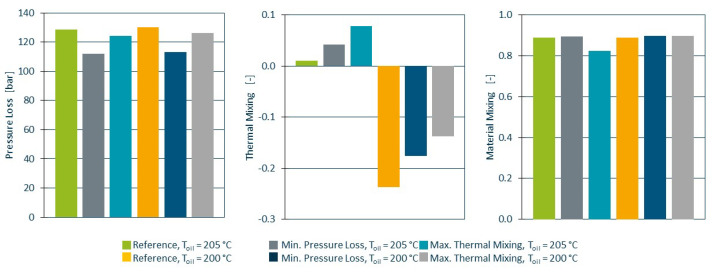
Pressure loss (**left**), thermal mixing (**centre**) and material mixing (**right**) for all simulations. Best results are seen for the higher oil temperature and optimisation for minimal pressure loss.

Figure 8 depicts the pressure loss (left), thermal mixing (centre) and material mixing (right) for the two different oil temperatures. Both the optimisation strategy for minimal pressure loss as well as thermal mixing result in reduced pressure loss and improved thermal mixing relative to the reference geometry, while material mixing was barely affected. The best overall result was observed for the optimisation for minimal pressure loss with an oil temperature 5 ${}^{\circ }$C above the melt temperature. In this scenario, material was removed from the mixing bars, therefore reducing the resistance to the flow as well as heating caused the additional shear. As a result, the pressure loss was reduced from 129 bar to 112 bar, while $e_{thermal}$ improved from 0.010 to 0.042 and $e_{conc}$ barely changed (0.888 to 0.894).

**Figure 9:**
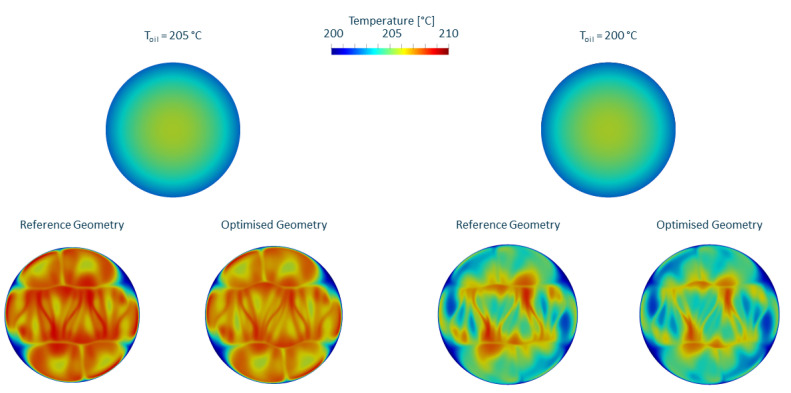
Thermal mixing for reference geometry and geometry optimised for pressure loss for an oil temperature 5 ${}^{\circ }$C above melt temperature (**left**) and at melt temperature (**right**).

**Figure 10:**
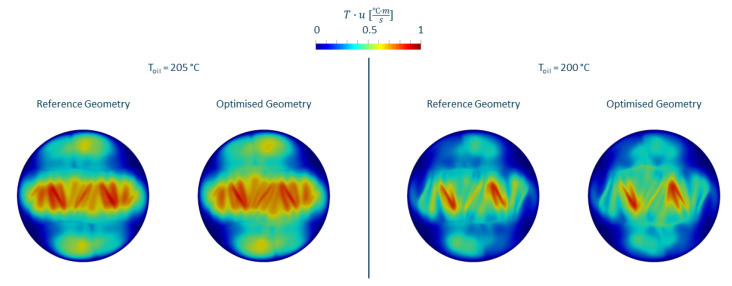
Temperature profile behind the mixer scaled for velocity for reference geometry and geometry optimised for pressure loss for an oil temperature 5 ${}^{\circ }$C above melt temperature (**left**) and at melt temperature (**right**).

The temperature profiles before and after mixing for both the reference and the mixer geometry optimised for minimal pressure loss are displayed in Figure 9; the left side shows the the higher oil temperature and the right side shows the oil temperature at melt temperature. Looking at the temperature profiles in isolation, it appears that the characteristic number for thermal mixing $e_{thermal}$ decreased for the cooler oil temperature because the effect size of the cooling channels outweighs the effect size of the mixing bars. For the in-depth interpretation of this result, we have to employ the Graetz number. The Graetz number Gr defines the thermal regime in a domain, where small numbers (<1) indicate an equilibrium and the melt near the cylinder wall takes on the wall temperature and large numbers (>100) indicate that convection is the dominant thermal transport mechanism, the melt temperature increases linearly with the distance traveled and the wall temperature is irrelevant [35]. It is defined as

(20) $$Gr=\frac{u\cdot H^{2}}{D_{T}\cdot L},$$

where *H* and *L* denote the height and length of the flow channel, respectively. For a throughput of 100 kg/h, a thermal diffusivity of $1.1997\times 10^{-7}$ $m^{2}/s$ and a flow channel length of 120 mm and a distance between mixing bars of 10 mm, the Graetz number is greater than 200. A different way to visualise this is seen in Figure 10, where the temperature profile (scaled to [0, 1]) at the outlet has been multiplied by the local velocities (scaled to [0, 1]), thus indicating enthalpy convection downstream. It is apparent that, at these high throughputs, the residence time of the melt in the mixer is not long enough for significant heat exchange to occur.

During the optimisation for either minimal pressure loss or maximal thermal mixing, the algorithm in both cases removed material from the mixer. To optimise for minimal pressure loss, the algorithm removed steel mainly towards the centre of the flow channel, where the impact of the thus reduced shear rate and flow resistance is the highest. This is consistent with previous findings, where optimisations of X-type mixers found a positive correlation between the number of mixing bars (i.e., reduction in total cross-section available for melt flow) and the pressure loss [10,36]. The results are also in agreement with [25] with respect to the changes to the geometry under the constraint of additive manufacturability. In addition, the thermal mixing improved, partly due to the reduction in the core temperature due to reduced viscous dissipation along the mixing bars [28], although these changes are small compared to the fluctuations and measurement uncertainties in the extrusion process. For thermal mixing on the other hand, the material removal was less concentrated in one location and a lot of material was removed closer to the cylinder wall as well. The removal of material here also explains the reduction in pressure loss as a secondary observation. Overall, the algorithm removed 6.7% of the static mixer geometry for minimal pressure loss and 12.3% for maximal thermal homogenisation. While the optimisation for thermal mixing was numerically successful, a closer investigation of the changes in the geometry found causes for concern regarding mechanical failure and leakage in locations not preemptively secured by the skeleton structure. For example, in some locations the mixer geometry surrounding a skeleton structure that was protecting an oil channel was completely removed and while this geometry is technically possible, the risk of failure was determined to be too high.

Based on these results, the reference geometry as well as the geometry obtained by optimisation for minimal pressure loss with an oil temperature 5 ${}^{\circ }$C above the melt temperature (hereinafter referred to as *optimised*) were chosen for manufacture and experimental evaluation.

### 3.2. Validation Trials

**Figure 11:**
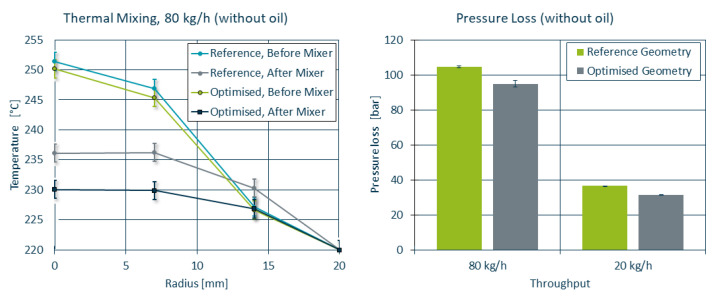
Thermal homogenisation and pressure loss of the reference and optimised mixer geometries. Note that the error bars in the temperature curves refer to the uncertainties in the temperature sensors as they outweigh the standard deviations in the recorded data.

The extruder setup used led to an increased temperature profile (see Figure 11 left), where the core of the melt reached temperatures as high as 250 ${}^{\circ }$C. While both static mixers managed to reduce the core temperature and improve overall homogeneity, the maximum core temperature for the optimised mixer was approximately 6 ${}^{\circ }$C lower compared to the reference mixer due to the decreased shear heating at the mixing bars. The pressure loss (see Figure 11 right) was reduced by 10% for the throughput of $80\frac{\mathrm{kg}}{h}$ (104.6 bar to 94.9 bar) and by 15% for $20\frac{\mathrm{kg}}{h}$ (36.45 bar to 31.3 bar).

**Figure 12:**
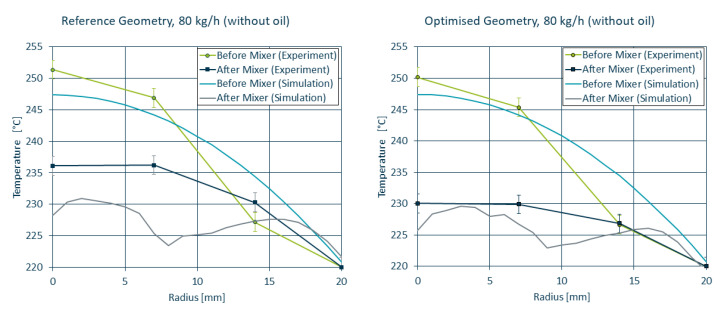
Simulated and measured temperature curves before and after the static mixer for the reference (**left**) and optimised (**right**) geometry.

For a direct comparison between simulation and experiment, the initial and boundary conditions of the extruder were recreated for OpenFOAM, corresponding to a melt temperature of 220 ${}^{\circ }$C with a $T_{max}$ = 250 ${}^{\circ }$C. The temperature profiles measured in the lab trials are in acceptable agreement with the temperature profiles obtained by means of simulation (see Figure 12). While the simulation tends to overestimate the cooling in the reference mixer geometry, it correctly predicts the improved homogeneity for the optimised mixer compared to the reference mixer.

**Figure 13:**
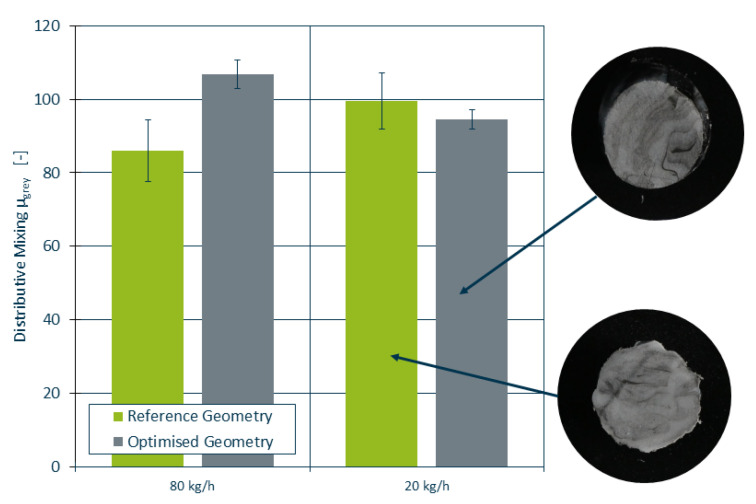
Distributive mixing capabilities of the reference and optimised mixer. High values of $\mu_{grey}$ are indicative of improved mixing.

During the trials, extrudate samples were taken, prepared and polished and subsequently photographed. An analysis of the colour distribution using ImageJ [37] and a *t*-test were performed to determine the statistical significance of the data. Two exemplary samples as well as the statistical analysis are shown in Figure 13. The *t*-test did not reject the null-hypothesis, suggesting no significant changes in the distributive mixing between the mixer geometries.

In Table 2, the results of the simulation as it was performed during the optimisation, the simulation using the initial and boundary conditions as observed during the lab trials and the data measured during the lab trials are compared side by side. Both of the simulations come to similar conclusions regarding the 13% reduction in pressure loss and 3 ${}^{\circ }$C reduction in maximum temperature at the outlet compared to their reference counterpart. While the lab trials measured a lower pressure loss reduction of only 9% and a larger temperature reduction of 6 ${}^{\circ }$C, these results are still in acceptable agreement. The material mixing was not affected in either of the cases.

**Figure 14:**
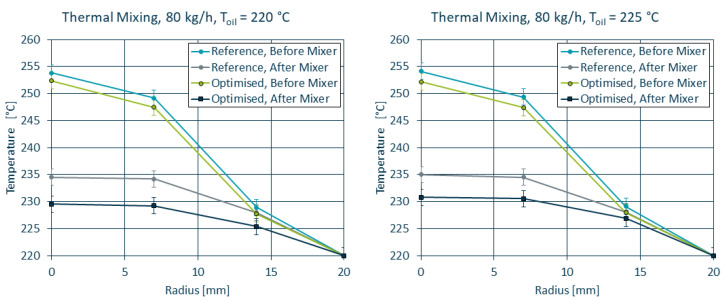
Temperature profiles of the reference and optimised geometries for both oil temperatures.

In addition, trials were run using the oil channels for additional cooling of the melt, once at the nominal melt temperature and once at a temperature 5 ${}^{\circ }$C higher than the nominal melt temperature. The results are depicted in Figure 14. As it was the case with the numerical results, the influence of the oil channels on the temperature curves was negligible at this high throughput. Compared to the trials run without the active cooling by the oil, the difference between the reference and optimised geometry was reduced by 1–2 ${}^{\circ }$C, putting the results closer to the results obtained by means of simulation.

## 4. Discussion

The mixer geometries obtained by the automatic optimisation algorithm fulfilled the SLM constraints and required only minimal post-processing and general user input to obtain a printable and usable static mixer geometry. While the approach taken is quite similar to the approaches taken by Langelaar et al. [25,38,39], there are a few key differences. In their research, the focus is on structural optimisation and their code is also able to insert support structures and determine the best printing direction [39]. The algorithm presented here is not as sophisticated, but has its own advantages. First of all, because the additive manufacturability is checked in each iteration, the computational cost has to be very low. In addition, the features described by Langelaar et al. are not required for this specific use case, as the printing direction can be easily determined as the direction of extrusion and the static mixer geometry has to be manufacturable without additional support structures, as the main reason to use AM in this context is the integration of the oil channels in locations that are not accessible after printing (e.g., to remove those support structures). In this regard, the strategy for the optimisation of static mixers is closer to the approach taken by Leary et al. [40], where the added support structures are an inherent part of the final geometry. However, the algorithm presented still requires improvement in future research. The update of the immersed boundary field as described in Equation (14) is highly sensitive to the relaxation parameters, which also need to be modified if the throughput changes or different optimisation targets are used at the same time.

Both the optimisation strategy for minimal pressure loss and maximal thermal homogeneity were able to minimise their target functions. Furthermore, the other quality criteria (pressure loss, thermal mixing, material mixing) also improved even if they were not the focus of the optimisation strategy itself. While the optimisation for minimal pressure loss also led to a secondary improvement in the thermal mixing, the optimisation for maximal thermal mixing also resulted in a slight reduction in pressure loss. The latter is to be expected, as the algorithm has a tendency to remove more cells than it adds, and a reduction of the mixing bar size is associated with a decrease in flow resistance, resulting in reduced pressure loss. Another reason lies in the way the target function for thermal homogeneity (Equation (13)) is formulated. In the current form, the individual optimisation target of each cell at the outlet is to get as close as possible to the target temperature, which is set at the desired melt temperature. As a result, the algorithm is incentivised to minimise the shear heating in the static mixer, which is achieved by a reduction of the mixing bar size. Unfortunately, the current algorithm only checks for local manufacturability. It does not automatically account for mechanical stability as this is handled via the skeleton structure. The optimisation for thermal mixing has shown that the skeleton structure used in this case was not sufficient in order to ensure a manufacturable geometry. In addition, the optimised geometry also worked for off-design throughputs, as shown in the validation trials. A different application for this algorithm can be the optimisation of the overall length of the mixer, which has been shown to be another significant factor [10,28].

The experimental results in the validation trials confirm the results obtained by means of CFD. The simulations tended to underestimate the cooling effect of the static mixer (3 ${}^{\circ }$C compared to the 4–6 ${}^{\circ }$C measured in the validation trials, depending on the oil temperature). Even without the utilisation of the temperature control channels, the static mixers were able to both cool and homogenise the plastic melt. These results should be seen in the context of the previous research published in [26,27,28], where the performance of the SLM-compatible static mixer was compared against a commercially available static mixer. They found that their SLM-compatible static mixer geometry improves the ratio of thermal mixing and pressure loss by 50% and enables cooling of the meld by up to 10 ${}^{\circ }$C. The initial geometry in the optimisation presented here was the result of that research, which implies that the method presented in this paper enables further optimisation compared to commercially available static mixers. Unfortunately, due to the low residence time at high throughputs, the thermal homogeneity could not be further improved by the oil channels. The temperature profiles after the mixer were the same regardless of the oil temperature, when accounting for measurement uncertainties. Future research should therefore focus on optimisation specifically for operating points at lower throughputs, where the chances for active cooling with the oil channels are improved. The material mixing did not fundamentally change between both mixer geometries, which is to be expected as the geometries were not different enough to change the distributive mixing behaviour.

One of the main advantages of the method presented is the reduced time (computational as well as skilled labour) for this method. While conventional optimisation methods need to run and analyse a minimum number of simulations and subsequently need to manually adjust the geometry and then re-simulate (or manufacture and validate in trials), and even automatic genetic optimisation algorithms need to evaluate a large amount of design points [3,8] the method presented only requires two to three simulations in total, i.e., initialisation, optimisation and an optional re-initialisation for evaluation purposes, resulting in less than 36 h of total run time. In addition, a lot less user input is required between the individual simulation runs, freeing the person performing the optimisation up to complete other tasks in the meantime. Therefore, automatic optimisation using Lagrange multipliers is a lot more efficient than manual and even simulation-assisted optimisation.

Future research should focus on the further development of the optimisation and the immersed boundary update algorithm. As the current IB update routine tends to favour the removal of solid cells instead of the addition of new solid cells, the extent to which new kinds of geometries can be created is very limited. In addition, a version where the skeleton structure can be moved to modify the position of the oil channels could enable the design of completely new structures. This future algorithm might take inspiration from [7], where a parameterised version of a static mixer modified the mixing geometry outside of a rigid geometry range. Also, an extension that checks the suitability for manufacture with conventional methods such as machining, assembly from sheets or casting instead of AM would be a useful addition and would broaden the field of applications for this method. A development of a user interface could improve industry adaptation of this method.

In the method presented, the initial geometry needs to already be suitable for AM, as the geometry would otherwise be dramatically altered to fit SLM constraints in the first iteration. To counteract that, coupling the algorithm to a routine that helps to improve the geometry’s suitability for AM prior to optimisation, e.g., by adding transitional elements to horizontal bars to make them manufacturable will expand the fields of application for this optimisation method.

## 5. Conclusions

While static mixers are widely used for the thermal and material homogenisation of melt in extrusion, previous attempts at optimisation for reduced pressure loss and improved mixing had to be performed by hand and human experience. A new automatic optimisation method based on the open source software OpenFOAM was developed. With the utilisation of immersed boundary methods and the implementation of new target functions for improved homogeneity in the pre-existing routine, *adjointShapeOptimizationFoam*, along with an additional algorithm checking the suitability for additive manufacturing and adjusting the geometry during run-time, was presented.

The new algorithm was used to optimise an existing static mixer based on an X-type geometry with integrated oil channels maximising the heat exchange between oil and melt, enabling simultaneous cooling and mixing. Based on the results of these simulative optimisations, the best candidates were manufactured using SLM and experimental trials were run. Experimental validation has shown that with this optimisation algorithm, a pressure loss reduction of 10–15% was achieved and the core melt temperature was reduced by another 6 ${}^{\circ }$C compared to the reference, improving the thermal homogenisation as well. These results are in good agreement with the results predicted by the simulations. While the main advantage of this method is the rapid optimisation taking the operating point into account, the trials also showed positive results in off-design operating points. This allows the low-cost design and manufacture of individualised static mixers.

## Figures and Tables

**Table 1:** Boundary Conditions for CFD simulations.

	Inlet	Outlet	Wall	Inlet Oil	Outlet Oil
u	$u_{melt}$	zeroGradient	noSlip	$u_{oil}$	zeroGradient
$p_{norm}$	zeroGradient	$1\times 10^{-5}\frac{m^{2}}{s^{2}}$	zeroGradient	zeroGradient	$1\times 10^{-5}\frac{m^{2}}{s^{2}}$
*T*	see Equation (15)	zeroGradient	$T_{melt}$	$T_{oil}$	zeroGradient
*c*	see Equation (16)	zeroGradient	zeroGradient	zeroGradient	zeroGradient
$u_{a}$	(−1 0 0)	see Equation (12)	noSlip	zeroGradient	zeroGradient
$p_{a,norm}$	zeroGradient	see Equation (11)	zeroGradient	zeroGradient	$1\times 10^{-5}\frac{m^{2}}{s^{2}}$
$T_{a}$	see Equation (15)	see Equation (13)	see Equation (13)	$T_{oil}$	zeroGradient

**Table 2:** Comparison between the results obtained by simulation and lab trials. Changes labeled as *Reduction* refer to the change with respect to their counterpart before the numerical optimisation.

	Simulative Optimisation	Validation (CFD)	Validation (Lab Trials)
$T_{melt}$	200 ${}^{\circ }$C	220 ${}^{\circ }$C	220 ${}^{\circ }$C
$T_{max,inlet}$	205 ${}^{\circ }$C	248 ${}^{\circ }$C	250 ${}^{\circ }$C
Reduction Δp	13% at $100\frac{\mathrm{kg}}{h}$	13% at $80\frac{\mathrm{kg}}{h}$	9% at $80\frac{\mathrm{kg}}{h}$
Reduction $T_{max,outlet}$	3 ${}^{\circ }$C	3 ${}^{\circ }$C	6 ${}^{\circ }$C
Material mixing	almost identical across reference and optimised geometries		

## Data Availability

Data available on request.

## Abbreviations

The following abbreviations are used in this manuscript:

AM	Additive Manufacturing
CFD	Computational Fluid Dynamics
IB	Immersed Boundary
IKV	Institute for Plastics Processing
SLM	Selective Laser Melting
WLF	Williams, Landel and Ferry
